# Face mask use and viral load in patients with mild symptoms of COVID-19

**DOI:** 10.31744/einstein_journal/2024AO0495

**Published:** 2024-10-23

**Authors:** Murilo Soares Costa, Claudia Regina Lindgren Alves, Flávio Guimarães da Fonseca, Hugo Itaru Sato, Raissa Prado Rocha, Alex Fiorini de Carvalho, Karine Lima Lourenço, Nathalia Sernizon Guimarães, Elaine Leandro Machado, Santuza Maria Ribeiro Teixeira, Unaí Tupinambás, Ricardo Hiroshi Caldeira Takahashi

**Affiliations:** 1 Universidade Federal de Minas Gerais Faculdade de Medicina Belo Horizonte MG Brazil Graduate Program in Infectious Diseases and Tropical Medicine, Faculdade de Medicina, Universidade Federal de Minas Gerais, Belo Horizonte, MG, Brazil.; 2 Universidade Federal de Minas Gerais Faculdade de Medicina Department of Pediatrics Belo Horizonte MG Brazil Department of Pediatrics, Faculdade de Medicina, Universidade Federal de Minas Gerais, Belo Horizonte, MG, Brazil.; 3 Universidade Federal de Minas Gerais Vaccine Technology Center Belo Horizonte MG Brazil Vaccine Technology Center, Universidade Federal de Minas Gerais, Belo Horizonte, MG, Brazil.; 4 University of Surrey Surrey United Kingdom University of Surrey, Guildford, Surrey, United Kingdom.; 5 Universidade Federal de Minas Gerais Escola de Enfermagem Department of Nutrition Belo Horizonte MG Brazil Department of Nutrition, Escola de Enfermagem, Universidade Federal de Minas Gerais, Belo Horizonte, MG, Brazil.; 6 Universidade Federal de Minas Gerais Faculdade de Medicina Department of Preventive and Social Medicine Belo Horizonte MG Brazil Department of Preventive and Social Medicine, Faculdade de Medicina, Universidade Federal de Minas Gerais, Belo Horizonte, MG, Brazil.; 7 Universidade Federal de Minas Gerais Faculdade de Medicina Department of Internal Medicine Belo Horizonte MG Brazil Department of Internal Medicine, Faculdade de Medicina, Universidade Federal de Minas Gerais, Belo Horizonte, MG, Brazil.; 8 Universidade Federal de Minas Gerais Department of Mathematics Belo Horizonte MG Brazil Department of Mathematics, Universidade Federal de Minas Gerais, Belo Horizonte, MG, Brazil.

**Keywords:** SARS-CoV-2, Masks, N95 respirators, Respiratory protective devices, Infectious, Communicable diseases, Viral load, COVID-19, Coronavirus disease

## Abstract

**Objective:**

Previous studies indicated that face masks reduce the probability of infection by SARS-CoV-2 but did not examine the relationship between SARS-CoV-2 viral load and mask usage. This study analyzed this relationship.

**Methods:**

This cross-sectional study evaluated patients admitted to a public Emergency Care Unit in Belo Horizonte, MG, Brazil, between October 2020 and March 2021. Convenience samples were recruited during the study period. Adult patients with COVID-19 symptoms were invited to participate after being examined by a physician according to the Emergency Care Unit routine. This study included 441 adults with mild symptoms diagnosed with COVID-19 via RT-PCR, who were tested until the 14^th^ day after symptom onset. Participants were interviewed about mask usage, oropharyngeal swabs were collected, and the SARS-CoV-2 viral load was measured using RT-PCR. The difference between the SARS-CoV-2 viral load in the groups of individuals who regularly used and did not use masks was subjected to a hypothesis test.

**Results:**

Of the patients who had swabs collected 1-5 days after symptom onset, 64.9% of those not using masks regularly and 45.5% of those using masks regularly presented a high SARS-CoV-2 viral load. This difference was statistically significant (p=0.0073). Considering only the patients who had swabs collected 4-8 days after symptom onset, 44.1% of those not using masks regularly and 30.8% of those using masks regularly presented a high SARS-CoV-2 viral load; this difference was statistically significant (p=0.0364).

**Conclusion:**

When people who regularly wear a face mask contract COVID-19, they have a significantly lower probability of having a high viral load of SARS-CoV-2 compared to those who do not regularly wear masks.

## In Brief

Previous studies have shown that face masks reduce the risk of SARS-CoV-2 infection but not the relationship between viral load and mask usage. This study analyzed 441 adults with mild COVID-19 admitted to a public Emergency Care Unit in Belo Horizonte, Brazil. Participants were interviewed about mask usage and SARS-CoV-2 viral load was measured using RT-PCR. Regular mask users had significantly lower viral loads than non-regular mask users (p=0.0073).

## Highlights

■ Probability of infection with a high SARS-CoV-2 viral load depends on mask-wearing habits.
■ The higher probability of infection with high viral load occurs for individuals that don't use mask regularly [47.3%.79.8%].
■ For individuals who use masks regularly, the probability is 38.6%.52.1%.

## Graphical abstract

**Figure:**
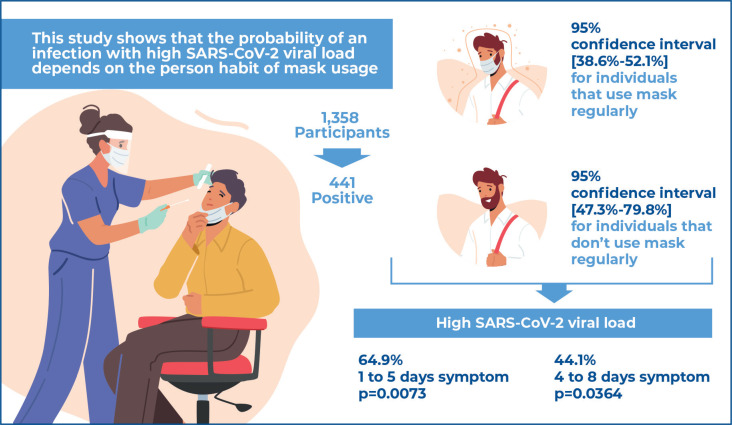

## INTRODUCTION

SARS-CoV-2 is a virus that invades the human body mainly through the respiratory tract, causing COVID-19.^(1)^ Shortly after the 2020-2022 pandemic was declared, a global discussion on disease prevention with non-pharmaceutical measures began. Among these, the use of face masks by the general population stands out because of their known effectiveness in preventing airborne infectious diseases.^(2,3)^

As pointed out by Howard et al.,^(4)^ logistical and ethical reasons preclude conducting randomized controlled trials to assess the effectiveness of masks in reducing the community transmission of COVID-19. Therefore, other types of evidence should be examined. Laboratory studies have shown that surgical face masks significantly reduce the spread of SARS-CoV-2 viral particles by infected individuals, both in droplets and aerosols,^(5)^ and that consumer-grade face masks have a filtering efficiency similar to those used in medical procedures.^(6)^ Other studies have examined the incidence of COVID-19 in populations and correlating it with adherence to mask use,^(7-9)^ concluding that using masks significantly reduce disease transmission.

Two observational studies analyzed the effectiveness of masks in preventing COVID-19. One study examined secondary disease transmission in households in 124 families with at least one confirmed COVID-19 case in Beijing, China,^(10)^ showing that facemasks were 79% effective at preventing disease transmission. In a study in Hiroshima, Japan,^(11)^ 820 close contacts of individuals diagnosed with COVID-19 were examined, and 16.4% of those not wearing masks and 7.1% of mask users were infected.

## OBJECTIVE

To analyze the association between face mask usage and SARS-CoV-2 viral load in patients with COVID-19 confirmed using RT-PCR. To the best of our knowledge, no similar studies have been published.

## METHODS

This cross-sectional study evaluated patients admitted to a public Emergency Care Unit (ECU) in Belo Horizonte, Minas Gerais, Brazil, between October 2020 and March 2021. During this period, Belo Horizonte recorded the two highest peaks in the number of COVID-19 cases: in early January 2021 and March 2021.

This study was part of a large research project approved by the *Universidade Federal de Minas* Gerais Committee (CAAE: 35074720.3.0000.5149; #4.249.706). Convenience samples were collected from the ECU during the study period. Only patients over 18 years of age who had presented mild or moderate symptoms compatible with COVID-19 in a previous examination carried out by the Emergency Unit team were invited to participate. "Mild symptoms" was defined as presenting with few signs and symptoms, while "moderate symptoms" was defined as presenting with several signs and symptoms, including dyspnea, but without needing oxygen mask use. Among the invited patients, only those who signed an Informed Consent Form and agreed to participate in the study were included.

A total of 1,358 patients were included in this study; 441 patients with RT-PCR results positive for SARS-CoV-2 were included in the present analysis. Participants were interviewed regarding sociodemographic aspects and clinical manifestations, such as the number of days from the onset of symptoms and adherence to COVID-19 prevention measures, including the use of face masks.

The number of days from the onset of symptoms to the moment of swab collection for testing varied from 1 to 14 days. The variable use of masks was obtained by asking, "When you leave home, do you wear a mask covering your mouth and nose?" The answer options were (i) every time, (ii) most of the time, (iii) sometimes, (iv) rarely, or (v) never. Participants who answered (i) or (ii) were classified as "regular use of mask" (RM), and the ones who answered (iii), (iv), or (v) were classified as "not regular use of mask" (NM).

The presence of SARS-CoV-2 RNA was tested using RT-PCR (pool testing) of nasopharyngeal swab samples from all eligible participants. As described by Costa et al.,^(12)^ if the pool was positive, the samples were analyzed individually to determine which samples were positive, and their cycle threshold (Ct) was measured. Patients who tested positive were classified into the high (Ct <20) and non-high (20 <Ct ≤37) SARS-CoV-2 viral load groups. Patients with Ct values of >37 were considered negative for SARS-CoV-2. It should be noted that the viral load is a continuously changing value, and there are no reports of a turning point indicating a sudden change in a patient's clinical condition when the Ct value falls below that value. Therefore, choosing a boundary between the Ct values indicating a high and low SARS-CoV-2 viral load was arbitrary. The threshold Ct of 20 was chosen because it was the smallest integer value that classified one-third of the participants as having a high SARS-CoV-2 viral load.

Data were entered by two researchers using Microsoft Excel software. Two other researchers conducted consistency assessments to ensure data authenticity and reliability. Data analysis was performed using Matlab Statistics and Machine Learning Toolbox from Matlab^®^ R2021a. Hypothesis tests assuming a binomial distribution of the event "high SARS-CoV-2 viral load" and significance level α=0.05 were performed to assess the statistical significance of the difference between the proportions of high SARS-CoV-2 viral load infections observed between RM and NM patients. Analysis was performed assuming independence between the outcomes of different patients. The confidence intervals were determined by assuming binomial distributions at a confidence level of 95%.

## RESULTS

Among the 441 RT-PCR-positive patients, 55.3% were female, 71.0% declared themselves black or brown, and 41.3% were aged between 30 and 49 years (range: 18-90 years). Table 1 shows the number of patients who responded to each mask usage option.

**Table 1:** Number of patients positive for SARS-CoV-2 that answered each option of mask usage

Mask usage answer	Number of patients	Mask usage classification
Every time	322	Regular (RM)
Most of the time	65	
Sometimes	42	Not regular (NR)
Rarely	10	
Never	2	

RM: regular use of mask; NM: not regular use of mask.

Table 2 shows the mean and standard deviation of the Ct values of the RT-PCR tests in four groups of patients: (i) RM patients with high SARS-CoV-2 viral load, (ii) RM patients with low SARS-CoV-2 viral load, (iii) NM patients with high SARS-CoV-2 viral load, and (iv) NM patients with low SARS-CoV-2 viral load. Lower RT-PCR cycle threshold (Ct) values indicate a higher viral load. In this study, it was assumed that a Ct value above 20 indicated a low SARS-CoV-2 viral load and a Ct value below 20 indicated a high SARS-CoV-2 viral load. A total of 387 patients (87.8%) reported regular mask use (RM patients), whereas 54 (12.2%) reported non-regular use of mask (NM patients). A total of 166 patients (37.6%) were diagnosed with a high SARS-CoV-2 viral load and 275 patients (62.4%) were diagnosed with a low SARS-CoV-2 viral load.

**Table 2:** Relationship between face mask usage and SARS-CoV-2 viral load

Mask usage	SARS-CoV-2 viral load	Mean Ct (standard deviation)	n (%)	
Regular (RM)	High	16.9 (1.9)	138 (35.7)	387 (100)
Regular (RM)	Not high	26.4 (4.7)	249 (64.3)	
Not regular (NM)	High	17.2 (1.8)	28 (51.9)	54 (100)
Not regular (NM)	Not high	27.0 (5.0)	26 (48.2)	

RM: regular use of mask; NM: not regular use of mask.

The number of days from symptom onset to the date of swab collection for the 441 patients who tested positive for SARS-CoV-2 ranged from 1 to 14 days. The median number of days was 5, with the 25^th^ percentile being 3 days and the 75^th^ percentile being 7 days.

**Figure 1:**
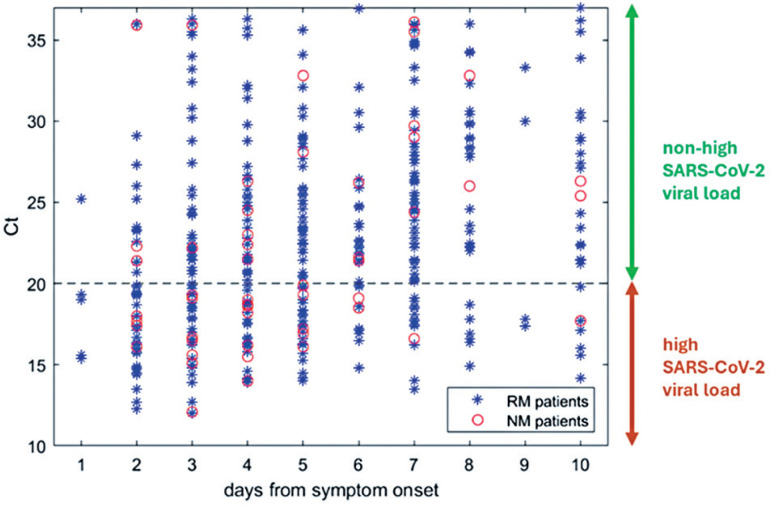
SARS-CoV-2 viral load indicator *versus* the number of days from symptom onset for each patient that had the swab collected until ten days after symptom onset

Figure 1 shows the SARS-CoV-2 viral load *versus* the number of days from symptom onset for each patient from whom swabs were collected until 10 days after symptom onset. Higher Ct values indicate lower SARS-CoV-2 viral loads. Although some patients had the swab collected from 11 to 14 days after symptom onset the NM patient who waited longer for testing had the swab collected 10 days after symptom onset.

RM patients (regular use of mask) are indicated by blue asterisks and NM patients (non-regular use of masks) are indicated by red circles.

The SARS-CoV-2 viral load is expected to decrease as the number of days from symptom onset to the date of swab collection increases, increasing the Ct. To classify the patients into relatively homogeneous groups, swabs were collected in five-day windows, ranging from 1-5 days to 10-14 days after symptom onset. These windows are overlapping, so the first window refers to patients who had their swabs collected between one day after symptom onset and five days after symptom onset, while the second window refers to patients who had their swabs collected between two days after symptom onset and six days after symptom onset, and so on.

Table 3 shows the results of the analysis of the groups of RM and NM patients in each time window. The first column indicates the beginning and end of each 5-day time window. The second column shows the maximum likelihood estimate of the probability of RM patients who had swabs collected within a given time window having a high SARS-CoV-2 viral load, indicated by P(high|RM), while the third column shows the 95%CI of that probability. The fourth column shows the maximum likelihood estimate of the probability of NM patients who had swabs collected within that time window having a high SARS-CoV-2 viral load, as indicated by P(high|NM), and the fifth column shows the 95%CI of that probability. The last column shows the p-value associated with the hypothesis that the probability of NM patients having a high SARS-CoV-2 viral load was greater than that of RM patients having a high SARS-CoV-2 viral load, under the assumption of a binomial distribution for the groups of patients within each time window. Statistical significance was set at p <0.05, with a significance level of 5%.

**Table 3:** Probabilities of having high SARS-CoV-2 viral loads in patients who regularly used face masks and those who did not in each time window

Time window (days)	P(high|RM)	95%CI	P(high|NM)	95%CI	p value P(high|NM) >P(high|RM)
1-5	0.453	[0.386-0.521]	0.649	[0.475-0.798]	0.0073
2-6	0.427	[0.364-0.491]	0.619	[0.456-0.764]	0.0054
3-7	0.337	[0.281-0.397]	0.548	[0.387-0.702]	0.0024
4-8	0.307	[0.249-0.370]	0.441	[0.272-0.621]	0.0364
5-9	0.287	[0.224-0.356]	0.381	[0.181-0.616]	0.1325
6-10	0.233	[0.168-0.309]	0.235	[0.068-0.499]	0.2317
7-11	0.210	[0.142-0.292]	0.167	[0.021-0.484]	0.1480
8-12	0.215	[0.123-0.335]	0.200	[0.005-0.716]	0.2400
9-13	0.222	[0.101-0.392]	0.333	[0.008-0.906]	0.2522
10-14	0.200	[0.091-0.357]	0.333	[0.008-0.906]	0.1733

Table 4 shows the number of individuals in each situation (RM patients with a high SARS-CoV-2 viral load, RM patients with a low SARS-CoV-2 viral load, NM patients with a high SARS-CoV-2 viral load, and NM patients with a low SARS-CoV-2 viral load) for each time window.

**Table 4:** Number of individuals in each situation for each time window

Time window (days)	Number of individuals			
	RM / H	RM / L	NM / H	NM / L
1-5	101	122	24	13
2-6	105	141	26	16
3-7	91	179	23	19
4-8	74	166	15	19
5-9	55	136	8	13
6-10	35	113	4	13
7-11	26	96	2	10
8-12	14	49	1	4
9-13	8	27	1	2
10-14	8	31	1	2

RM: regular use of mask; NM: not regular use of mask.

**Figure 2:**
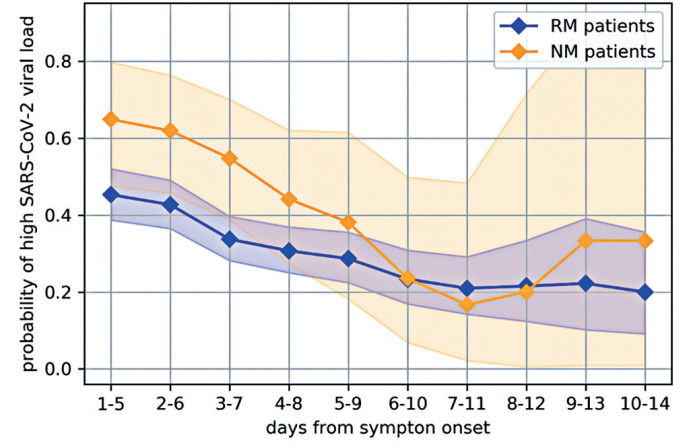
Maximum likelihood estimates of the probabilities of high and non-high SARS-CoV-2 viral loads across groups of patients in all time windows

Figure 2 shows the maximum likelihood estimates of the probability that a patient positive for SARS-CoV-2 had a high viral load. Each point estimate with its corresponding 95%CI was calculated considering the results for all patients that had swabs collected within a given time window, as indicated in the horizontal axis.

**Figure 3:**
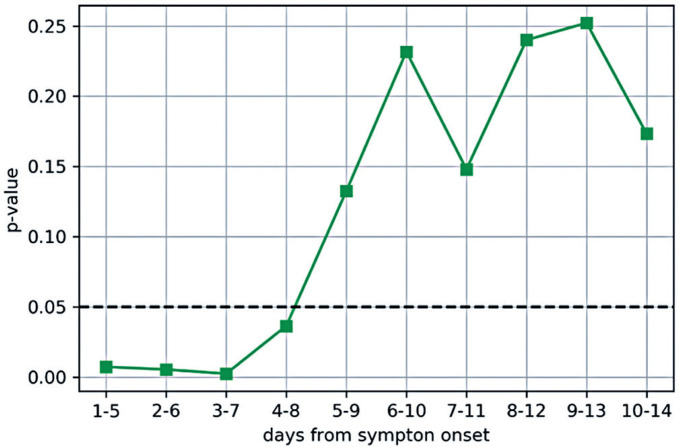
P-values of the hypothesis that the probability of patients not regularly using face masks to have a high SARS-CoV-2 viral load is greater than that of patients who regularly use masks to have a high SARS-CoV-2 viral load

Next, we examined the alternative hypothesis that the probability of an NM patient presenting with high SARS-CoV-2 viral load is higher than that of an RM patient against the null hypothesis stating that there is no evidence, up to a significance of 5%, that the probability of presenting with a high SARS-CoV-2 viral load is different for RM and NM patients. The test was conducted separately for groups of patients whose swabs were collected within each time window after symptom onset. The p-values associated with the alternative hypotheses are presented in the last column of table 3 and figure 3. Figure 3 shows the p-values for the hypothesis that the probability of NM patients having a high SARS-CoV-2 viral load is greater than that of RM patients having a high SARS-CoV-2 viral load, considering the groups of patients defined for each time window. Statistical significance was set at p<0.05, with a significance level of 5%.

Figure 3 shows that when considering the time windows of 1-5 days, 2-6 days, 3-7 days, and 4-8 days after symptom onset, the p-values associated with the hypothesis that the probability of presenting high SARS-CoV-2 viral load in NM patients is higher than the probability of presenting high SARS-CoV-2 viral load in RM patients were <0.05, leading to hypothesis acceptance for these time windows. The null hypothesis cannot be rejected for the time windows starting after the 4^th^ day from symptom onset.

It should be noted that in all time windows, the confidence intervals of the maximum likelihood estimates of probabilities of RM patients presenting with high SARS-CoV-2 viral load were much narrower than those of NM patients. This was due to the small number of NM patients included in this study.

## DISCUSSION

The use of face masks as a preventive measure to contain the spread of the COVID-19 epidemic was proposed even before there were specific studies on the effect of such protective devices against SARS-CoV-2, based on the existence of well-established evidence of the effectiveness of mask use as protection against various airborne diseases.^(2-4)^To address this knowledge gap, numerous studies have been conducted throughout the COVID-19 pandemic to investigate different aspects of the effects of face mask use in the context of this disease. The effect of face masks in avoiding infection by SARS-CoV-2 has been examined in several studies.^(7-11)^ Other studies^(5,6)^ showed that masks significantly reduce the exposure of individuals to SARS-CoV-2 in droplets and aerosols, although they do not eliminate the risk of infection. It has also been found that the SARS-CoV-2 viral load is correlated with disease severity.^(13)^

This study builds on the existing research on mask usage by examining a previously unexplored area: the relationship between mask use and an individual's viral load after contracting SARS-CoV-2. The present study shows that face mask usage is associated with a lower probability of having a high SARS-CoV-2 viral load in symptomatic patients, mainly when the first symptoms emerge.

Figure 2 and table 3 illustrate the trajectories of the maximum likelihood estimates for the probability of high viral load in RM and NM patients. These trajectories and their respective confidence intervals can be explained as follows. Clinical evidence suggests that a patient's viral load typically peaks during the initial days of infection and then declines as they recover. Consequently, the probability of RM and NM patients having a high SARS-CoV-2 viral load should decrease as the time since symptom onset increases. This trend was evident in the first five time windows (1-5, 2-6, 3-7, 4-8, and 5-9 days after symptom onset). In addition, the last four time windows considered here (7-11, 8-12, 9-13, and 10-14 days after symptom onset) were characterized by a widening of the confidence intervals due to the decreasing number of patients in those intervals.

Therefore, the data analyzed in this study support conclusions only for the first four time windows (1-5, 2-6, 3-7, and 4-8 days after symptom onset), as the hypothesis tests found p<0.05. The other later time windows did not indicate significant differences due to these factors: (i) the viral load decreases as the time from symptom onset increases, the NM patients and RM patients have viral loads that become more similar, and (ii) as the number of NM patients decrease, the confidence intervals for their probability of having high viral load become wider. Both factors tend to increase the p-value associated to the hypothesis test.

### Analysis methodology

It should be noted that grouping the patients according to the number of days between symptom onset and swab collection, considering five-day time windows for defining the groups, involves a trade-off between the imprecision caused by including patients in different stages of disease evolution in the same group and that caused by the small number of RM or NM patients in a given group. A smaller time window would define more homogeneous groups but would lead to wider confidence intervals for the same number of participants. The choice of five-day windows considered this trade-off; this is the smallest window size that still led to reasonably separate confidence intervals.

Another issue regarding the data analysis methodology is the arbitrary choice of Ct=20 as the separation threshold between the conditions of high and low viral load. It should be noted that the same analysis, when performed with thresholds of Ct=19 and 21, led to the same general results reported here. However, when Ct ≤18, the results will differ because the number of patients with a high SARS-CoV-2 viral load will decrease. This will cause the confidence intervals for the estimates of the probability of NM patients having high viral loads to become too wide such that the corresponding confidence intervals for RM patients will fall within those intervals, and the p-values of the hypothesis test will be >0.05. In contrast, for a threshold of 22 or higher, the number of patients with a high SARS-CoV-2 viral load will increase significantly, and then the number of patients with a low SARS-CoV-2 viral load will decrease for both RM and NM patients. This will cause the groups of RM and NM patients to become more similar and the hypothesis test in some time windows will not achieve p<0.05 for the alternative hypothesis. Nevertheless, the hypothesis tests still indicated that the probability of RM patients having high SARS-CoV-2 viral loads was lower than that of NM patients with high SARS-CoV-2 viral loads for at least two of the first three time windows for all thresholds up to Ct=25. In summary, choosing a separation threshold of Ct=20 for defining high and low viral loads was not critical.

### Limitations

It is also important to emphasize that many other variables besides mask usage can affect the SARS-CoV-2 viral load, such as the sample collection technique, storage condition, patient immunity, and other pre-analytical, analytical, and post-analytical factors.^(13)^ In this sense, one limitation of the present study was that no other immunological or clinical aspects that impact SARS-CoV-2 viral loads were obtained from the participants. Another limitation is that the participants who wore masks were not asked about the type of mask; therefore, no inference could be made concerning that variable.

## CONCLUSION

The main conclusion of this study was that regular face mask use was associated with a lower probability of high SARS-CoV-2 viral load in the first eight days after symptom onset among individuals who contracted COVID-19 than among those who did not regularly use face masks. Our findings reinforce the importance of using face masks as a public health measure to reduce the spread of SARS-CoV-2 in the community.
